# MicroRNA-27 Prevents Atherosclerosis by Suppressing Lipoprotein Lipase-Induced Lipid Accumulation and Inflammatory Response in Apolipoprotein E Knockout Mice

**DOI:** 10.1371/journal.pone.0157085

**Published:** 2016-06-03

**Authors:** Wei Xie, Liang Li, Min Zhang, Hai-Peng Cheng, Duo Gong, Yun-Cheng Lv, Feng Yao, Ping-Ping He, Xin-Ping Ouyang, Gang Lan, Dan Liu, Zhen-Wang Zhao, Yu-Lin Tan, Xi-Long Zheng, Wei-Dong Yin, Chao-Ke Tang

**Affiliations:** 1 Institute of Cardiovascular Research, Key Laboratory for Atherosclerology of Hunan Province, Medical Research Center, Hunan Province Cooperative Innovation Center for Molecular Target New Drug Study, University of South China, Hengyang, Hunan, China; 2 Laboratory of Clinical Anatomy, University of South China, Hengyang, Hunan, China; 3 Department of Pathophysiology, University of South China, Hengyang, Hunan, China; 4 Department of Biochemistry and Molecular Biology, The Libin Cardiovascular Institute of Alberta, Cumming School of Medicine, The University of Calgary, Health Sciences Center, Hospital Dr NW, Calgary, Alberta, Canada; Northeast Ohio Medical University, UNITED STATES

## Abstract

Atherosclerotic lesions are lipometabolic disorder characterized by chronic progressive inflammation in arterial walls. Previous studies have shown that macrophage-derived lipoprotein lipase (LPL) might be a key factor that promotes atherosclerosis by accelerating lipid accumulation and proinflammatory cytokine secretion. Increasing evidence indicates that microRNA-27 (miR-27) has beneficial effects on lipid metabolism and inflammatory response. However, it has not been fully understood whether miR-27 affects the expression of LPL and subsequent development of atherosclerosis in apolipoprotein E knockout (apoE KO) mice. To address these questions and its potential mechanisms, oxidized low-density lipoprotein (ox-LDL)-treated THP-1 macrophages were transfected with the miR-27 mimics/inhibitors and apoE KO mice fed high-fat diet were given a tail vein injection with miR-27 agomir/antagomir, followed by exploring the potential roles of miR-27. MiR-27 agomir significantly down-regulated LPL expression in aorta and peritoneal macrophages by western blot and real-time PCR analyses. We performed LPL activity assay in the culture media and found that miR-27 reduced LPL activity. ELISA showed that miR-27 reduced inflammatory response as analyzed *in vitro* and *in vivo* experiments. Our results showed that miR-27 had an inhibitory effect on the levels of lipid both in plasma and in peritoneal macrophages of apoE KO mice as examined by HPLC. Consistently, miR-27 suppressed the expression of scavenger receptors associated with lipid uptake in ox-LDL-treated THP-1 macrophages. In addition, transfection with LPL siRNA inhibited the miR-27 inhibitor-induced lipid accumulation and proinflammatory cytokines secretion in ox-LDL-treated THP-1 macrophages. Finally, systemic treatment revealed that miR-27 decreased aortic plaque size and lipid content in apoE KO mice. The present results provide evidence that a novel antiatherogenic role of miR-27 was closely related to reducing lipid accumulation and inflammatory response via downregulation of LPL gene expression, suggesting a potential strategy to the diagnosis and treatment of atherosclerosis.

## Introduction

Atherosclerosis is one of the major causes leading to mortality of dysfunctional cardiovascular events in developed countries. The atherosclerotic lesions are mostly characterized by the transformation of macrophages to foam cells through uptake of lipoprotein-derived cholesterols, which secrete various inflammatory cytokines in the arterial intima[1], suggesting a critical role for macrophages in the development of atherosclerosis[2, 3].

Lipoprotein lipase (LPL), which is expressed and secreted by parenchymal cells in muscle and adipose tissues, could bind to the capillary endothelium and hydrolyze triglyceride (TG) core of circulating TG-rich chylomicrons (CM) and very low-density lipoproteins (VLDL) into free fatty acids (FFA) and glycerol[4]. LPL is considered as a protector against atherosclerosis by reducing atherogenic lipoproteins in a variety of tissues, such as the heart, skeletal muscle, adipose and aorta[5–7]. However, none of these actions is thought to involve macrophage LPL, which has been suggested to have a proatherogenic role[8]. The expression of macrophage LPL was enhanced markedly in the serum from familial hypercholesterolemia patients[9]. Furthermore, Azumi et al. demonstrated that the expression of LPL in macrophages has been associated with atherosclerotic lesions[10]. This conclusion has been substantiated by the observation that macrophage-specific expression of human LPL promotes the development of atherosclerosis in apoE knockout mice and rabbits[11, 12]. Interestingly, macrophage LPL knockout (MLPLKO) mice using cre-loxP gene targeting did not show any changes in plasma LPL activities or lipoprotein levels but had a decrease in cholesterol ester foam cell formation and diet-induced atherosclerosis[13]. Furthermore, our group and others revealed that the inhibition of macrophages-derived LPL expression has beneficial effects on lipid metabolism and inflammatory response [14–16], further supporting a proatherogenic role for macrophage LPL. One possible explanation for those properties of LPL expressed by macrophages is that LPL may contribute to the retention of atherogenic lipoproteins for subsequent cellular uptake and gene expression of inflammatory factors acting as a non-enzymatic molecular bridge between lipoprotein receptors and proteoglycans in subendothelial spaces[17]. Therefore, manipulating macrophage LPL to reduce lipid accumulation and inflammatory response has been an important therapeutic goal of atherosclerosis.

MicroRNAs (miRNAs), a group of endogenous non-coding RNAs of ~22 nucleotides, have been identified as important negative regulators at the posttranscriptional level[18–21]. Most recently, several miRNAs have been found to affect lipid metabolism and inflammatory response by us and others[14, 15, 22–24]. It has been proposed that two isoforms of the miR-27 family, miR-27a and -27b present in the macrophage cell lines, may participate in the initiation and progression of atherosclerosis as we recently reviewed[20]. MiR-27 was found to inhibit adipocyte differentiation that is closely associated with the onset of obesity[25, 26], and also participate in lipid metabolism in the liver[27]. Our group has previously demonstrated that miR-27a/b repressed the expression of endogenous LPL through binding directly to the LPL 3’UTR, and then affected the metabolism of cellular cholesterol in THP-1 macrophages[28]. In addition, miR-27a was detected with twofold lower expression to LPS-induced inflammatory response in the RAW264.7 cells[29], indicating that the targets of miR-27a are involved in inflammatory response. These findings suggest that miR-27 may have a close relationship with atherosclerosis. To date, however, it is still not clear whether miR-27 plays a role in the development of atherosclerosis through targeting macrophage LPL *in vivo*. In this study with gain- and loss-of-function experiments, we used artificial microRNA mimics/inhibitor or agomir/antagomir to investigate the roles of miR-27a/b in lipid metabolism and inflammatory response and atherosclerotic lesion formation *in vitro* or *in vivo*. Enhancing miR-27a/b function protected apoE KO mice from atherosclerosis as a result of obvious reduction of the expression of LPL, lipid uptake, and pro-inflammatory cytokine secretion, but inhibition of miR-27a/b function had the opposite effects.

## Materials and Methods

### Cell culture

Human monocytes (THP-1) and RAW 264.7 cells were obtained from the Cell Bank of the Chinese Academy of Sciences in Shanghai, China. THP-1 and RAW 264.7 cells were cultured in RPMI 1640 media (Sigma) containing 10% (v/v) fetal calf serum (FBS, Gibco BRL, America), 100 U/mL penicillin and 100 mg/mL streptomycin. To initiate differentiation into macrophages, THP-1 cells were induced in differentiation medium supplemented with 160 nmol/L phorbol-12-myristate 13-acetate (PMA, Sigma, America) for 24 h. All these cells were maintained in a humidified atmosphere containing 5% CO_2_ at 37°C.

### Animals and treatments

The 8-week-old male apoE KO mice were obtained from Nanjing CAVENS Biological Technology Co, Ltd. All mice were randomly allocated to 6 groups: miR-27a/b agomir group (AGa and AGb), miR-27a/b scrambled agomir negative control group (AG-NC), miR-27a/b antagomir group (ANa and ANb) and miR-27a/b scrambled antagomir negative control group (AN-NC). Each group was composed of fifteen mice. The mice were fed on Western-type diet (WTD, 21% fat, 0.2% cholesterol) and water until sacrificed, and maintained on a 12-h light-dark cycle. Experimental mice were injected with miRNA agomir/antagomir or their respective controls via tail vein (GuangZhou RiboBio. Co.) at a dose of 80 mg /kg wt in 0.2 ml saline once every four weeks. After 8 weeks, the mice were bled to death using 20% urethane anesthetized. All animal experiments were approved by the Animal Ethics Committee of the University of South China, and carried out according to the Guide for the Care and Use of Laboratory Animals published by the US National Institutes of Health (NIH Publication number 85–23, revised 2011).

### LPL activity

The activities of LPL secreted by macrophages in culture media were determined using the LPL kit as described previously[9]. The medium was supplemented with 0.5 U/ml heparin at the end of the incubation period, followed by detection of LPL activities in the medium. Levels of LPL activities were normalized to total cell proteins. In the reaction system, production of 1 μmol free fatty acid per mg protein per hour is expressed as one active unit.

### Real-time PCR analysis

Total RNA was isolated with TRIzol reagent (Invitrogen) from cells. Using SYBR Green detection chemistry, relative quantitative real-time PCR (RT-PCR) was performed on Light Cycler Run 5.32 Real-Time PCR System (Roche). Melting curve analyses of all real-time PCR products were performed and shown to produce a single DNA duplex. Quantitative measurements were determined using the ΔΔCt method. In each experiment, GAPDH was used as the internal control. The sequences of the real-time PCR primers were as follows: LPL, forward: 5’-GGGAGTTTGGCTCCAGAGTTT-3’, and reverse: 5’-TGTGTCTT CAGGGGTC CTTAG-3’; scavenger receptor A1 (SR-A1), forward: 5’-CTCGTGTTTGCAGT TCTCA-3’ and reverse: 5’-CCATGTTGCTCATGTGTTCC-3’; lectin oxidized low-density lipoprotein receptor (LOX-1), forward: 5’-TTACTCTCCATGGTGGTGCC -3’, and reverse: 5’-AGCTTCTTCTGCTTGTTGCC-3’; CD36, forward: 5’-GAGAACTGTTATGGGGCTAT-3’, and reverse: 5’-TTCAACTGGAGAG-GCAAAGG-3’; CXC chemokine ligand 16 (CXCL16), forward: 5’-ACTACACGACGTTCCAGCTCC-3’, and reverse: 5’-CTTTGTCCGAGGACAG TGATC -3’; GAPDH, forward: 5’-AACTTTGGCATTGTGGAAG G-3’, and reverse: 5’-ACACATTGGGGGTAGGAACA-3’.

### Western blot analysis

Cell lysates were extracted with RIPA buffer with protease inhibitor cocktail (Sigma). Protein (20 μg) loaded in each lane was subjected to SDS-PAGE (10% gels), and then transferred to a polyvinylidene difluoride (PVDF) membrane. After blocking with 5% skimmed milk for 4 h, PVDF membranes were incubated with a primary antibody overnight at 4°C, and then incubated with a secondary antibody for 2 h. The protein bands were detected using chemiluminescence immunoblotting detection system (Amersham Biosciences, USA). Western blot analysis was conducted as described previously [23, 24].

### Lipid analysis by high-performance liquid chromatography assays (HPLC)

Six hours after incubation with ox-LDL, THP-1 macrophages were transfected with miRNA mimic or inhibitor, followed by further incubation for 24 h. HPLC assay was conducted, as described previously, for the detection of free cholesterol and cholesterol ester[23]. Cells were detached in PBS supplemented with 1% EDTA (T4174; Sigma). The sterol was analyzed using an HPLC system (model 2790; Waters Corp.). A photodiode array detector equipped with a 4 μL cell was applied to detect the sterols (model 996; Waters Corp.). Analysis of cholesterol and cholesteryl esters was performed with acetonitrile-isopropanol 30:70 (v/v) after elution. Absorbance at 210 nm was detected. Total Chrom software from PerkinElmer was performed to analyze all Data.

### Cytokine detection with enzyme-linked immunosorbent assay (ELISA) assay

ELISA assay was performed to detect the levels of secreted pro-inflammatory cytokines. The concentrations of interleukin-1β (IL-1β), interleukin-6 (IL-6), monocyte chemoattractant protein 1 (MCP-1) and tumor necrosis factor α (TNF-α) in the supernatants were analyzed according to the manufacturer's instructions.

### Transfection of LPL short-interfering RNA (siRNA)

LPL siRNA was purchased from Santa Cruz Biotechnology (sense: GCAAAUUUGCCC UAAGGACTT, antisense: GUCCUUAGGGCAAAUUUGCTT). Control nonsilencing siRNAs were obtained from the Biology Engineering Corporation in Shanghai, China. Both LPL siRNA and control siRNA were transfected into THP-1 macrophages (2×10^6^ cells/well) using Lipofectamine 2000 (Invitrogen).

### Atherosclerosis analysis

The mice were perfused by cardiac puncture with 4% (w/v) paraformaldehyde to wash out blood from the heart and all vessels after euthanasia, and the surrounding fat and connective tissue were removed carefully from the aorta and heart, then observed by a stereomicroscope. Tissues including hearts, aortas, abdominal cavity macrophages and blood samples were collected for the further measurements. For en face analysis, the whole aorta was excised from the aortic arch to the common iliac artery and stained with oil-red O[30]. To analyze the atherosclerotic lesions in the aortic root, 5 μm frozen sections of the aortic root were prepared and also stained with hematoxylin-eosin (H&E) and oil red O[31]. Images of the sections were obtained with a microscope. Lesion areas were quantified using IMAGEPRO PLUS software.

### Statistical analysis

Data were presented as means ± SD from at least three independent experiments. All results between groups were analyzed by applying the one-way analysis of variance (ANOVA) and Student's t-test. A difference with P<0.05 was regarded as statistical significance. Statistical analyses were performed using SPSS 18.0 software.

## Results

### MiR-27a/b inhibits LPL expression in vitro and in vivo

**Fig 1:**
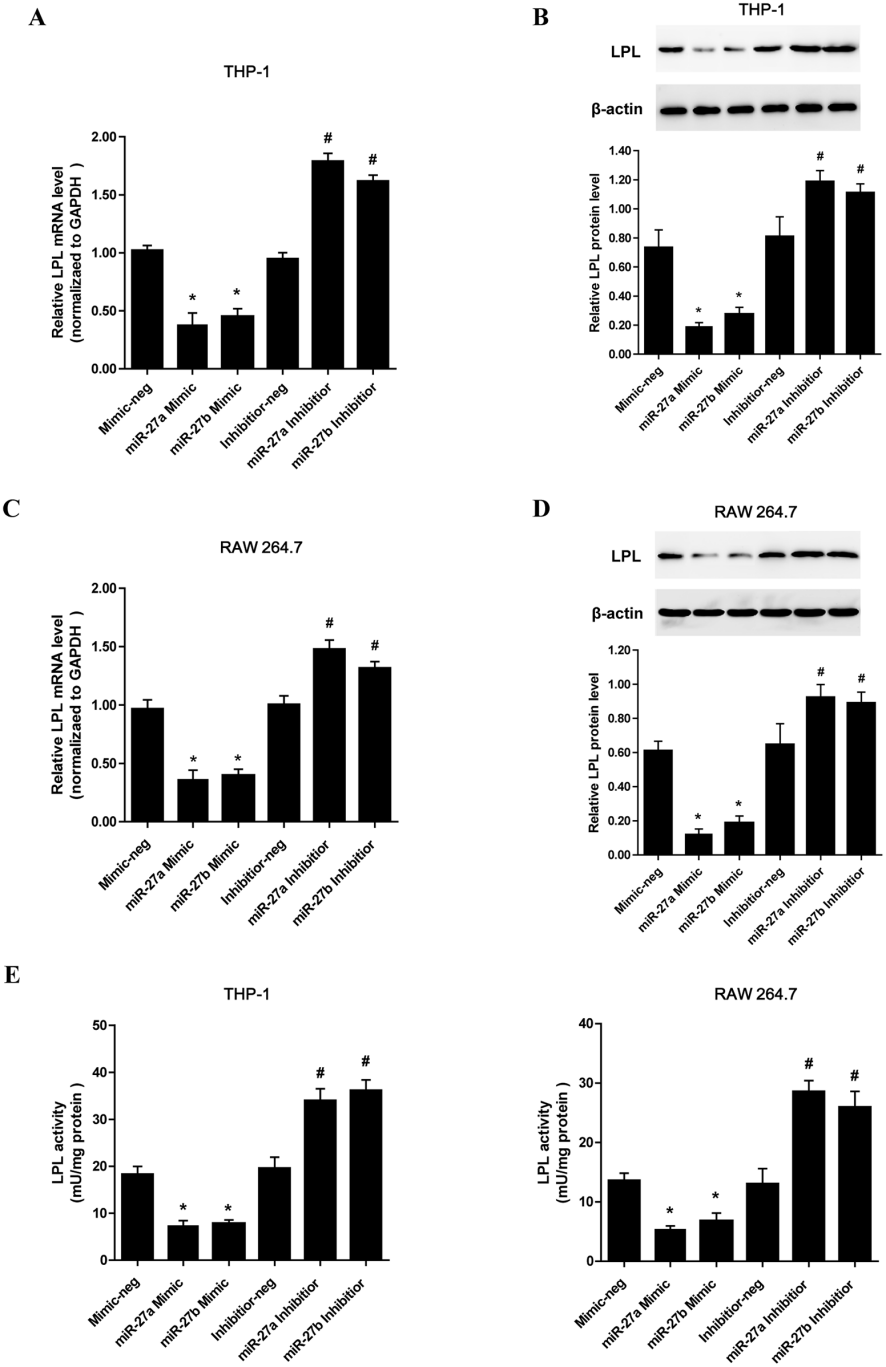
Effects of miR-27a/b on LPL expression and activities in various cell types. (A and B) LPL mRNA and protein levels were measured by RT-qPCR and western blot assay, respectively, in THP-1 cells transfected with miR-27 mimic or inhibitor. (C and D) LPL mRNA and protein levels were measured by RT-qPCR and western blot assay in RAW 264.7 cells transfected with miR-27 mimic or inhibitor, respectively. (E) Changes of LPL activities in the culture media of THP-1 macrophages and RAW 264.7 cells treated with miR-27a/b mimic or inhibitor, respectively. All results are expressed as mean±S.D. from three independent experiments. *P<0.05 vs. mimic-neg, #P<0.05 vs. inhibitor-neg.

Our group has previously demonstrated that miR-27a/b repressed the expression of endogenous LPL through binding directly to the LPL 3’UTR in THP-1 macrophages[28]. Consistently, the expression of LPL mRNA and protein in both THP-1 macrophages (Fig 1A and 1B) and RAW 264.7 cells (Fig 1C and 1D) was changed accordingly by miR-27a/b inhibitor and mimic. At the same time, the activity assay showed that LPL activities were decreased in the cells transfected with miR-27a/b mimic, but increased in the cells transfected with miR-27a/b inhibitor (Fig 1E). Taken together, our results have further established a role for miR-27a/b in the negative regulation of endogenous LPL expression in various macrophage cell types.

**Fig 2:**
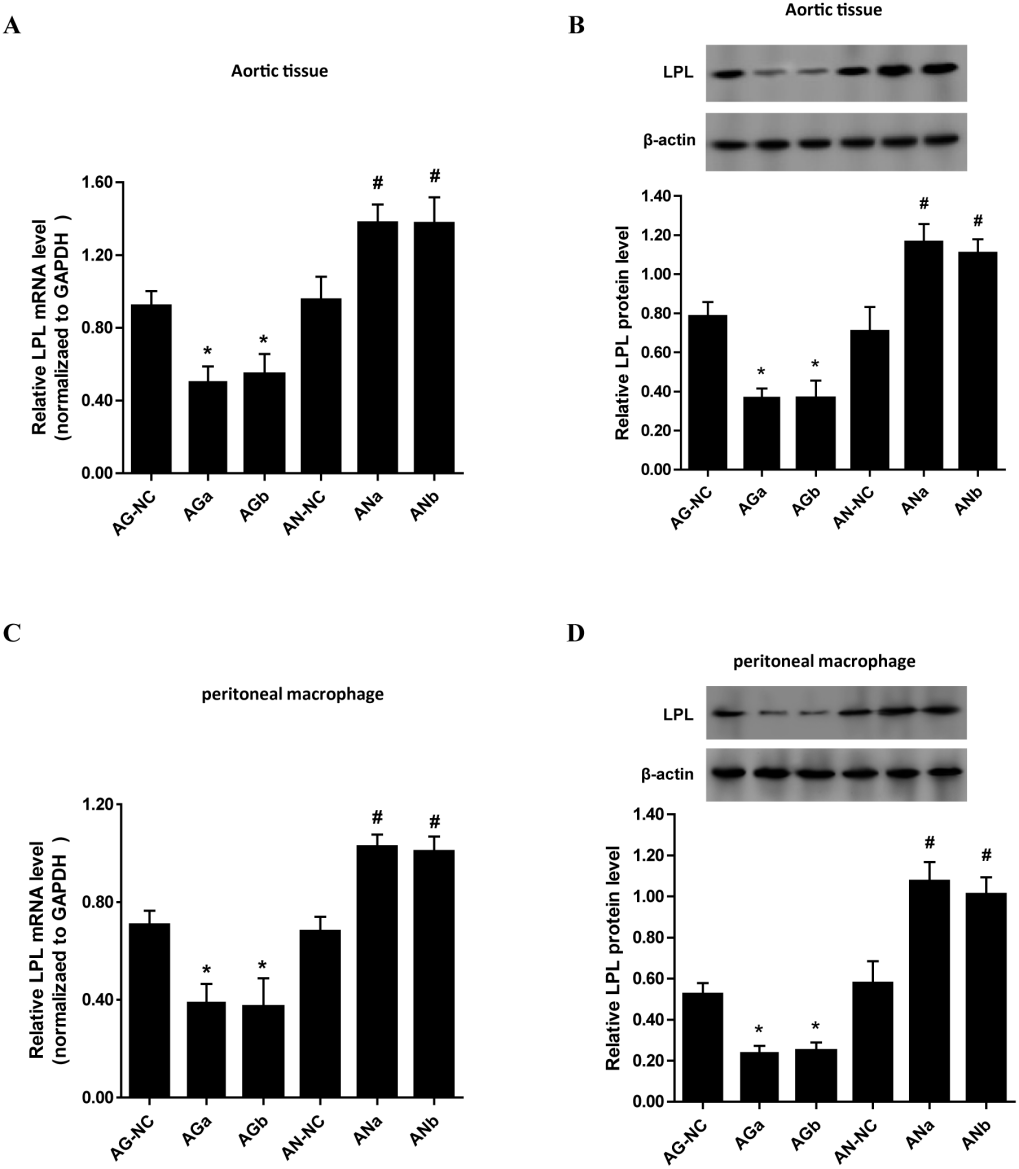
Effects of miR-27a/b on LPL expression in apoE KO mice. Eight-week-old male apoE KO mice (n = 10 mice per group) fed high-fat diet were given a tail vein injection with miR-27a/b agomir (AGa and AGb) or its scrambled agomir negative control (AG-NC), miR-27a/b antagomir (ANa and ANb) or its scrambled antagomir negative control (AN-NC). (A and B) LPL mRNA and protein levels in aortic tissues of apoE KO mice were measured by RT-qPCR, respectively. (C and D) LPL mRNA and protein levels in peritoneal macrophages of apoE KO mice were measured by RT-qPCR and western blot assay, respectively. All results are expressed as mean±S.D. *P<0.05 vs. AG-NC, #P<0.05 vs. AN-NC.

To investigate further the effects of miR-27a/b on LPL expression *in vivo*, we assessed LPL expression in tissue homogenate of isolated aortic roots in apoE KO mice treated with miR-27a/b agomir or antagomir or their respective scrambled controls. Compared with respective control groups, LPL expression levels of mRNA and protein were markedly down-regulated in miR-27a/b agomir-treated group, whereas up-regulated in miR-27a/b antagomir-treated group, in aortic roots of apoE KO mice (Fig 2A and 2B).

Furthermore, in peritoneal macrophages of apoE KO mice treated with miR-27a/b agomir, we found a significant reduction in LPL mRNA and protein levels. However, an increase in LPL expression was observed in miR-27a/b antagomir-treated mice (Fig 2C and 2D). These results illustrated that miR-27a/b inhibited macrophage LPL expression in apoE KO mice.

### MiR-27a/b reduces production of proinflammatory cytokines

**Fig 3:**
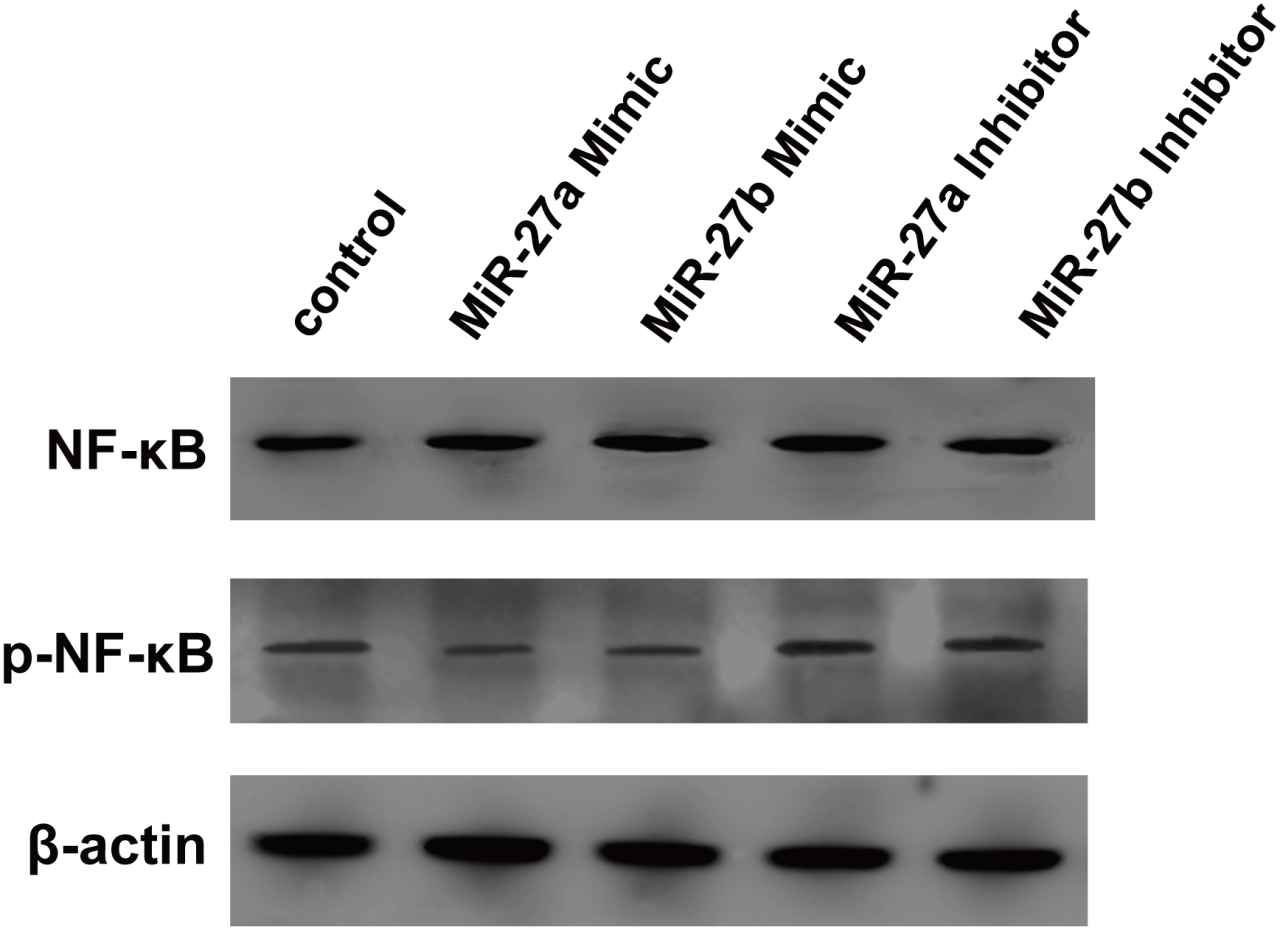
Effects of miR-27a/b on the expression of NF-κB in human THP-1 macrophages. Expression of NF-κB protein and its phosphorylation status was detected by western blot analysis in THP-1 macrophages treated with miR-27a/b mimic or inhibitor.

To investigate the functional consequences of miR-27a/b expression, we examined the production of pro-inflammatory cytokines in ox-LDL-stimulated THP-1 macrophages using ELISA. As shown in Table 1, the levels of IL-1β, IL-6, MCP-1, and TNF-α were significantly decreased by treatment of cells with miR-27a/b mimic. When cells were treated with miR-27a/b inhibitor, those cytokines were markedly increased. Both mimic and inhibitor control microRNAs had no effect on the secretion of the four pro-inflammatory cytokines (Table 1). These data suggest that transfection with miR-27a/b could negatively affect the inflammatory response of ox-LDL-stimulated THP-1 macrophages. NF-κB is a key inflammatory gene identified in THP-1 macrophages. We next examined the level of NF-κB phosphorylation using western blot analysis. As demonstrated in Fig 3, phosphorylation of NF-κB was decreased in response to miR-27a/b mimic treatment. On the contrary, its phosphorylation was increased by treatment of cells with miR-27a/b inhibitor.

**Table 1:** Effects of miR-27a/b on secretion of proinflammatory cytokines in ox-LDL- treated THP-1 macrophages.

	IL-1β (ng/L)	IL-6 (ng/mL)	MCP-1 (ng/L)	TNF-a (ng/L)
**control**	183.04±25.07	0.87±0.17	197.37±23.89	91.36±10.94
**Mimic-neg**	181.97±19.26	0.86±0.13	199.09±20.53	87.17±8.03
**miR-27a mimic**	78.66±16.14*	0.27±0.09*	102.56±12.40*	37.59±12.36*
**miR-27b mimic**	85.28±11.30*	0.35±0.08*	109.28±16.86*	40.86±8.33*
**Inhibitor-neg**	180.26±24.82	0.82±0.13	194.89±24.20	88.91±8.96
**miR-27a inhibitor**	230.25±26.17*	1.42±0.16*	241.70±37.79*	128.15±16.58*
**miR-27b inhibitor**	228.91±25.73*	1.38±0.10*	232.03±38.21*	124.57±17.94*
**miR-27a inhibitor+LPL-siRNA**	163.73±16.25^#^	0.85±0.18^#^	187.52±28.83^#^	78.79±11.84^#^
**miR-27b inhibitor+LPL-siRNA**	159.86±12.13^#^	0.80±0.14^#^	185.46±28.64^#^	77.57±13.04^#^
**LPL**	235.49±15.59*	1.76±0.15*	250.95±22.15*	140.75±21.22*
**LPL-siRNA**	73.79±12.93*	0.45±0.13*	104.84±19.66*	49.67±12.77*

The concentrations of inflammatory cytokines secreted in 24 h culture supernatants by stimulating THP-1 macrophages with various factors were measured by ELISA. All the results are expressed as mean±S.D. from three independent experiments.

*P<0.05 vs. control,

^#^P<0.05 vs. miR-27a/b inhibitor.

Given that miR-27a/b reduced proinflammatory cytokine production *in vitro*, we next measured the expression of plasma inflammatory cytokines in apoE KO mice to further explore the effects of miR-27a/b on inflammation *in vivo*. As shown in Table 2, treatment of apoE KO mice with miR-27a/b agomir significantly decreased the levels of IL-1β, IL-6, MCP-1 and TNF-α compared with those in control mice, consistent with the regulatory role of miR-27a/b in affecting inflammatory response *in vitro*.

**Table 2:** Effects of miR-27a/b on inflammatory cytokine production in the blood of apoE KO mice.

	IL-1β	IL-6	MCP-1	TNF-α
**AG-NC**	331.6±23.4	374.5±35.7	234.1±30.6	495.6±32.9
**AGa**	211.9±24.7*	225.0±27.5*	171.7±38.8*	271.7±30.6*
**AGb**	213.7±27.1*	226.6±31.0*	162.1±21.1*	274.6±23.1*
**AN-NC**	332.3±26.1	376.7±27.3	227.5±36.3	483.9±37.3
**ANa**	439.7±34.3^#^	451.4±36.7^#^	313.5±41.3^#^	583.9±44.3^#^
**ANb**	437.6±32.9^#^	457.6±46.2^#^	322.8±35.3	593.0±38.9^#^

Each group contained 10 mice. Unit: (pg/mL). AG-NC: miR-27a/b agomir negative control; AGa: miR-27a agomir; AGb: miR-27b agomir; AN-NC: miR-27a/b antagomir negative control; ANa: miR-27a antagomir; ANb: miR-27b antagomir; IL-1β: Interleukin-1β IL-6: Interleukin-6; MCP-1: monocyte chemotactic factor-1; TNF-α: tumor necrosis factor alpha. All the results are expressed as mean±S.D.

*P < 0.05 vs. AG-NC.

^#^ P < 0.05 vs. AN-NC.

### MiR-27a/b effects on lipid uptake involve scavenger receptors

**Fig 4:**
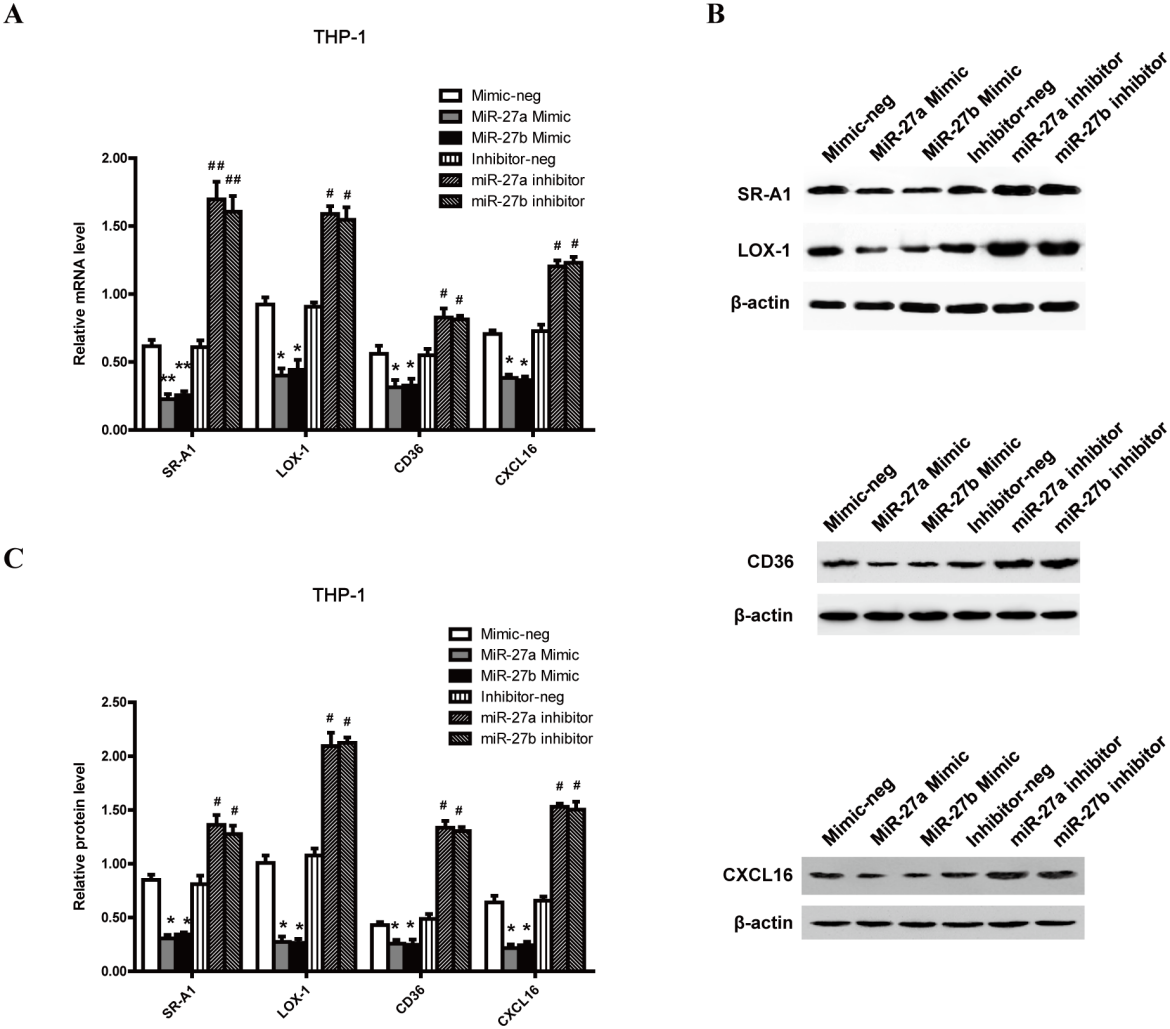
Effects of miR-27a/b on the expression of surface scavenger receptors. THP-1 macrophages were incubated with miR-27a/b mimic or inhibitor for 6 h, and then with ox-LDL for 24 h. SR-A1, LOX-1, CD36 and CXCL16 expression was determined after incubation with miR-27a/b mimic or inhibitor. (A) The relative mRNA levels of SR-A1, LOX-1, CD36 and CXCL16. (B and C) The protein levels of SR-A1, LOX-1, CD36 and CXCL16 were detected by western blot analysis. All results are expressed as mean±S.D. from three independent experiments. *P<0.05 vs mimic-neg, #P<0.05 vs inhibitor-neg, **P<0.01 vs mimic-neg, ##P<0.01 relative to inhibitor-neg.

It is well established that LPL can facilitate the cellular uptake of lipoproteins bound to the cell surface as a molecular bridge between lipoprotein and receptors[13, 32]. Given that miR-27a/b reduced LPL expression and attenuated the ability of lipid uptake in ox-LDL-stimulated THP-1 macrophages, we next detected the expression of associated scavenger receptors in ox-LDL-stimulated THP-1 macrophages to elucidate the mechanism underlying the effects of miR-27a/b on lipid uptake. Our results showed that miR-27a/b mimic significantly repressed the expression of SR-A1, LOX-1, CD36 and CXCL16 both at the mRNA and protein levels, whereas miR-27a/b inhibitor treatment had the opposite effects (Fig 4). These results suggest that scavenger receptor family may play an important role in the lipid uptake process regulated by miR-27a/b.

Considering the effects of miR-27a/b on lipid uptake in ox-LDL-stimulated THP-1 macrophages, we further investigated whether miR-27a/b affects the lipid levels in peritoneal macrophages of apoE KO mice. The levels of intracellular lipids were assessed using HPLC after miR-27a/b functions were manipulated. In Table 3, those peritoneal macrophages in miR-27a/b agomir-treated group showed a significant reduction in the levels of total cholesterol (TC), esterified cholesterol (CE) and free cholesterol (FC) compared with the negative control group. However, miR-27a/b inhibitor significantly enhanced the levels of those. Thus, these results suggest that miR-27a/b regulates not only cellular lipid uptake but also lipid composition in ox-LDL-stimulated macrophages.

**Table 3:** Effects of miR-27a/b on lipid accumulation in macrophages in abdominal cavity of apoE KO mice.

	TC	FC	CE	CE/TC (%)
**AG-NC**	521±20.74	188±24.52	333±26.03	63.92
**AGa**	412±30.05*	160±16.26*	252±26.21*	61.17
**AGb**	418±28.04*	162±17.52*	256±23.18*	61.24
**AN-NC**	523±27.57	184±18.04	339±29.37	64.82
**ANa**	609±21.78^#^	217±32.51^#^	392±26.31^#^	64.37
**ANb**	605±29.21^#^	220±32.60^#^	385±32.01^#^	63.64

Each group contained 10 mice. Unit: (mg/g protein). AG-NC: miR-27a/b agomir negative control; AGa: miR-27a agomir; AGb: miR-27b agomir; AN-NC: miR-27a/b antagomir negative control; ANa: miR-27a antagomir; ANb: miR-27b antagomir; TC: total cholesterol; FC: free cholesterol; CE: cholesterol ester. All the results are expressed as mean±S.D.

*P <0.05, vs AG-NC.

^#^ P <0.05, vs AN-NC.

### The role of LPL in the effect of miR-27a/b on the lipid uptake and proinflammatory cytokine secretion in THP-1 macrophages

**Fig 5:**
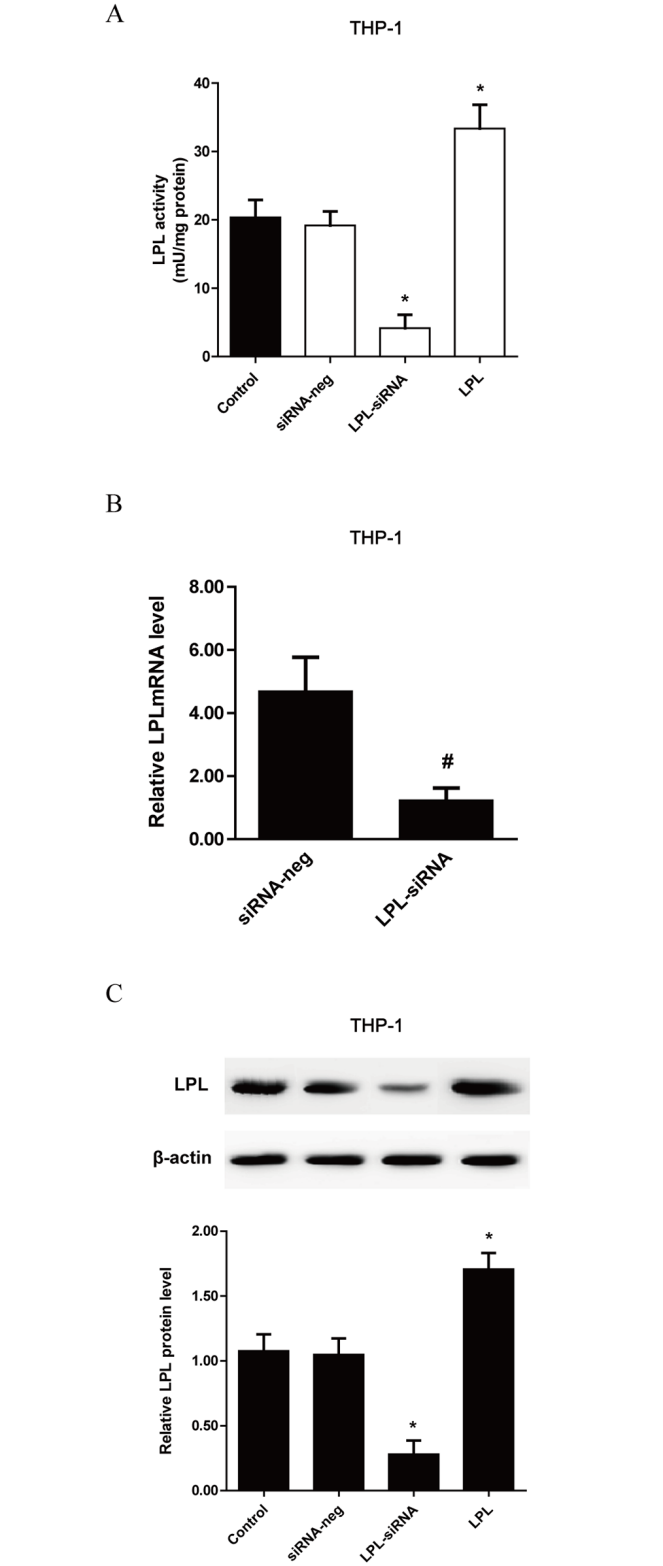
Effects of bovine LPL and LPL siRNA on the expressions and activities of LPL in ox-LDL-stimulated THP-1 macrophages. Confirmation of siRNA-induced knockdown of LPL activities (A), mRNA (B) and protein levels (C) after transfection with LPL-siRNA or an irrelevant control sequence (siRNA-neg) or treatment with and without bovine LPL in ox-LDL-stimulated THP-1 macrophages. All results are expressed as mean±S.D. from three independent experiments. *P<0.05 vs control. #P<0.05 vs siRNA-neg.

**Fig 6:**
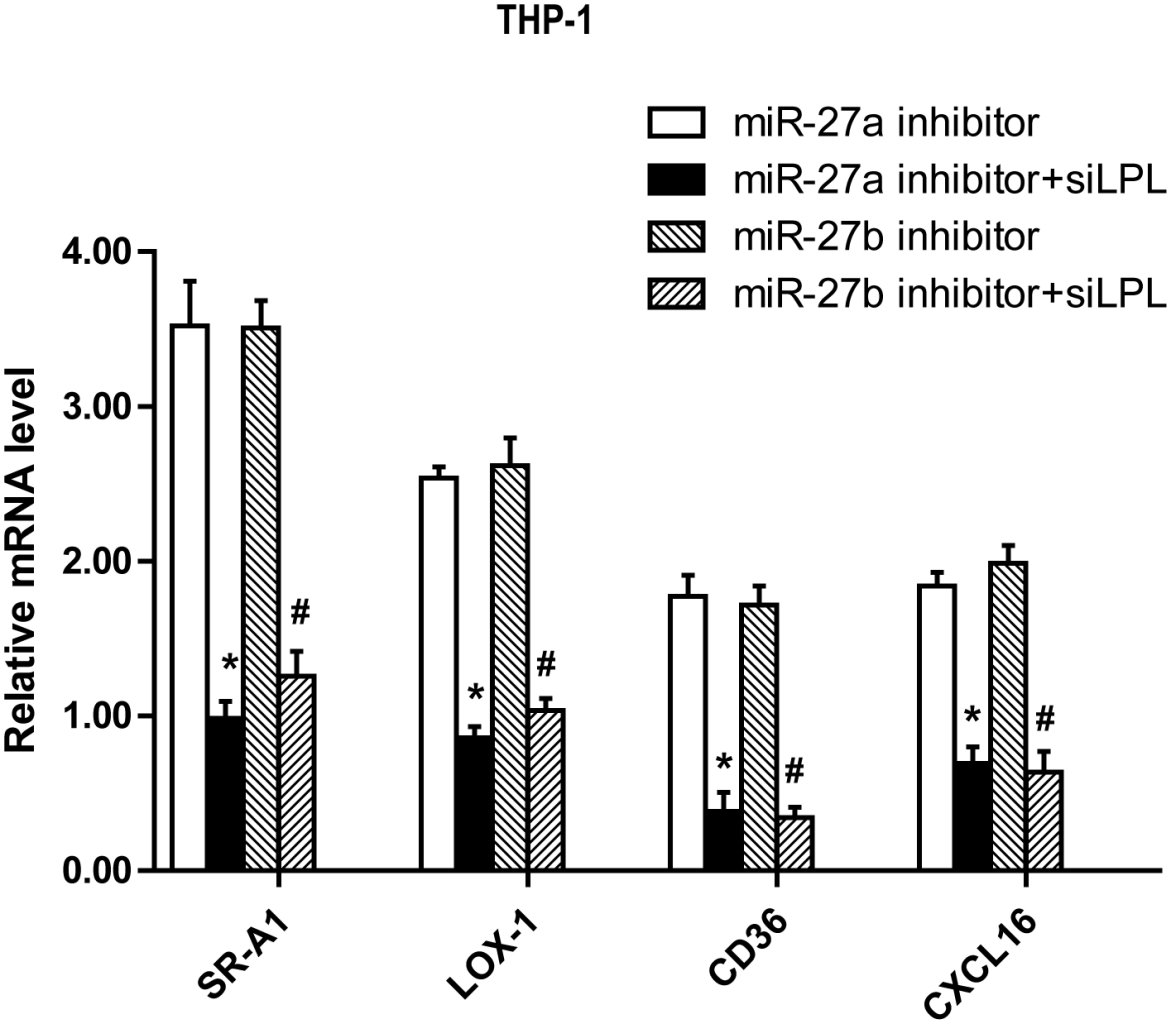
Effects of LPL on miR-27a/b-regulated expression of surface scavenger receptors in ox-LDL-stimulated THP-1 macrophages. The mRNA levels of SR-A1, LOX1, CD36 and CXCL16 were measured by RT-qPCR in THP-1 macrophages transfected with LPL siRNAs and then incubated with miR-27a/b inhibitors, respectively. All results are expressed as mean±S.D. from three independent experiments. *P<0.05 vs miR-27a inhibitor. #P<0.05 vs miR-27b inhibitor.

Considering the inhibitory effects of miR-27a/b on LPL expression and LPL effects on inflammatory response and lipid uptake, we wished to clarify further whether LPL functions as the target gene in miR-27a/b-mediated effects on lipids and proinflammatory cytokine secretion in ox-LDL-stimulated THP-1 macrophages. To do so, the expression of endogenous LPL was depleted or added using siRNA or bovine LPL (500 ng/ml), respectively. Our results showed that LPL activities and expression were modulated by LPL overexpression or siRNA. Knockdown of LPL expression by siRNA treatment efficiently repressed LPL activity (Fig 5A) and expression (Fig 5B and 5C). Furthermore, the effects of miR-27a/b inhibitor on scavenger receptor expression in THP-1 macrophages were attenuated by LPL siRNA (Fig 6). In addition, our results showed that the secretion of pro-inflammatory cytokines including TNF-α, MCP-1, IL-1β and IL-6 was obviously altered by treatment with bovine LPL or siRNA. As expected, LPL siRNA efficiently reduced the miR-27a/b inhibitor-induced secretion of these pro-inflammatory cytokines (Table 1). Taken together, our data suggest an essential role for LPL as a mediator of the biological effects of miR-27a/b in ox-LDL-treated THP-1 macrophages.

### MiR-27a/b affects plasma lipid profile

Because LPL hydrolyzes triglyceride and impacts macrophage lipid profile, we next examined the effects of miR-27a/b on plasma lipid levels in apoE KO mice. We found that miR-27a/b agomir-treated group significantly increased plasma TG levels, but decreased plasma TC and plasma LDL-C levels compared with mice treated with miR-27a/b agomir negative control. On the other hand, miR-27a/b antagomir decreased plasma TG levels, but increased plasma TC and plasma LDL-C levels in comparison with miR-27a/b antagomir negative control (Table 4). From these results, we conclude that miR-27a/b down-regulates plasma cholesterol levels in apoE KO mice.

**Table 4:** The Effects of miR-27a/b on body weight and plasma lipid profile in apoE KO mice.

	BW(g)	TG	TC	HDL-C	LDL-C
**AG-NC**	27.62±1.25	2.38±0.26	18.77±1.39	3.12±0.46	15.20±0.61
**AGa**	28.04±1.72	3.67±0.28*	14.03±1.57*	2.99±0.53	12.14±1.25*
**AGb**	27.72±1.20	3.59±0.37*	14.64±0.96*	2.92±0.49	11.70±1.58*
**AN-NC**	28.33±0.76	2.33±0.24	18.58±0.86	3.05±0.62	15.50±0.80
**ANa**	28.19±1.42	1.39±0.27^#^	23.11±1.04^#^	3.14±0.62	18.19±0.86^#^
**ANb**	27.87±0.80	1.30±0.25^#^	22.47±1.05^#^	3.23±0.54	18.59±0.91^#^

Plasma lipids from different experimental groups were measured with enzymatic methods. Unit: (mmol/L). AG-NC: miR-27a/b agomir negative control; AGa: miR-27a agomir; AGb: miR-27b agomir; AN-NC: miR-27a/b antagomir negative control; ANa: miR-27a antagomir; ANb: miR-27b antagomir; BW: body weight; TG: triglyceride; TC: total cholesterol; HDL-C: high density lipoprotein cholesterol; LDL-C: low-density lipoprotein cholesterol. All the results are expressed as means ± SD from the indicated numbers of male apoE KO mice in each group (n = 10 mice per group).

*P <0.05, vs AG-NC.

^#^P <0.05, vs AN-NC.

### MiR-27a/b inhibits aortic atherosclerosis

**Fig 7:**
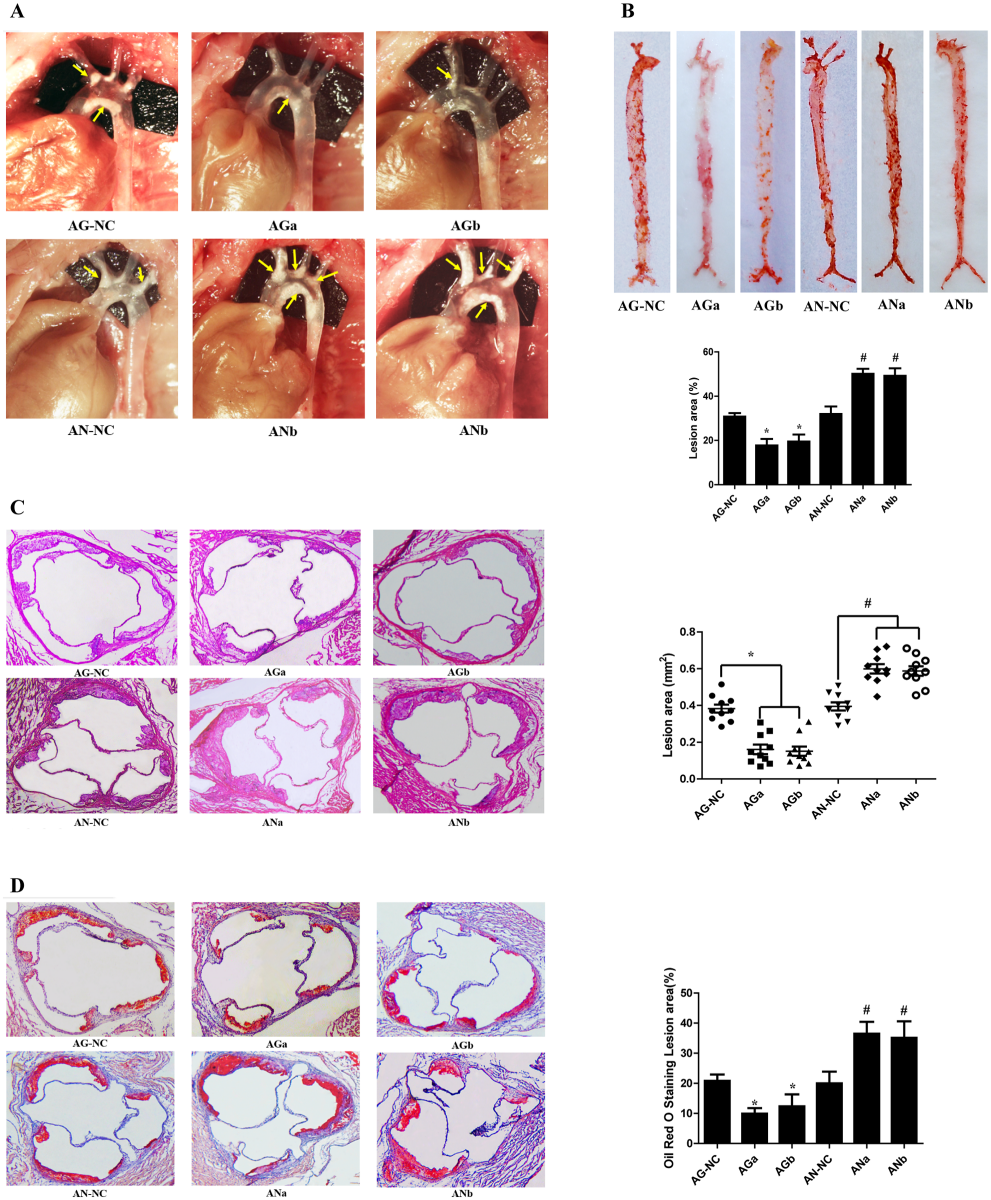
MiR-27a/b inhibits aortic atherosclerosis. Male 8-week-old apoE KO mice (n = 10 mice per group) fed high fat diet were given a tail vein injection with miR-27a/b agomir negative control (AG-NC), miR-27a/b agomir (AGa/AGb), miR-27a/b antagomir negative control (AN-NC) and miR-27a/b antagomir (ANa/ANb). (A) MiR-27a/b alleviates atherosclerotic plaque development in apoE KO mice. Plaques (arrows) in aortic arches and thoracic aortas of representative apoE KO mice are shown. (B) Effects of miR-27a/b on atherosclerotic lesion areas in apoE KO mice. Representative images and the quantification of atherosclerotic lesion areas in the en face analysis of the whole aorta with Oil red O staining. (C) Effects of miR-27a/b on aortic atherosclerotic lesions in aortic root in apoE KO mice. Representative micrographs were obtained from hematoxylin-eosin staining of cross-sections of proximal aorta in apoE KO mice. (D) Effects of miR-27a/b on the aortic sinus lesion areas in apoE KO mice. Characterization of aortic sinus atherosclerotic lesion areas was performed by Oil red O staining. Total Oil red O staining positive area was determined using IMAGEPRO PLUS Software. Images of representative sections from each group are accompanied by summarized bar charts. Each data point represents an individual animal. The horizontal lines denote the mean of each group. All results are expressed as mean±S.D.*P<0.05 vs AG-NC. #P<0.01 vs AN-NC.

It is known that atherosclerosis is characterized by the progressive plaque lesion. To further investigate the roles of miR-27a/b in the development of atherosclerosis in apoE KO mice fed high-fat diet, we measured the atherosclerotic lesion areas by an en face analysis of the whole aorta and the cross-sections of the aortic root. MiR-27a/b agomir reduced the number and size of plaques in the aortic arch and thoracic aorta region, but miR-27a/b antagomir had the opposite effects (Fig 7A). The atherosclerotic lesions of the whole aorta in en face were reduced in miR-27a/b agomir-treated group, but increased in miR-27a/b antagomir-treated group, when compared with their respective control mice (Fig 7B). The assessment of plaque in the hematoxylin- and eosin-stained cross-sections of the aortic root revealed that the plaque area in miR-27a/b agomir-treated mice was less than that those in mice treated with miR-27a/b scrambled agomir. Conversely, as shown in Fig 7C, the aortic root of miR-27a/b antagomir-treated mice contained more severe lesion than miR-27a/b scrambled antagomir-treated mice. Furthermore, severity of lipid deposition in the Oil red O stained cross-sections of the aortic root was alleviated in mice treated with miR-27a/b agomir, but enhanced in mice treated with miR-27a/b antagomir, compared with control groups (Fig 7D). Taken together, these results demonstrated that miR-27a/b inhibits the development of atherosclerotic lesions in apoE KO mice.

## Discussion

It is well known that the development of atherosclerosis, a chronic vascular disease, is closely associated with the subendothelial accumulation of macrophages and derived foam cells[33–36], one of the hallmarks of atherosclerosis. High expression of pro-inflammatory cytokines and excessive accumulation of lipids in activated macrophages have been shown to promote the progression of atherosclerotic lesions. Based on this, the novel measures to treat cardiovascular diseases driven by atherosclerosis have focused on regulating lipid metabolism and inflammatory response at the same time[37, 38]. Our current studies investigated the mechanisms underlying miR-27 effects on lipid metabolism and inflammatory response. We showed that miR-27 attenuated lipid accumulation and secretion of the pro-inflammatory cytokines, leading to a reduction in the development of atherosclerotic lesions in apoE KO mice, at least partially, by repressing the expression of LPL directly.

MiRNAs participate in the regulation of atherogenesis as shown in recent studies[39, 40], but the exact functions and mechanisms have not been fully elucidated. Our studies have shown that miRNAs acted as important modulators of lipid homeostasis and inflammation regulation for atherosclerosis[15, 22, 23, 28]. Recently, a notable role for miR-27 in the pathogenesis of atherosclerosis has been noticed and studied extensively, although previous studies about miR-27 were more involved in the field of tumor research. Many pieces of research have shown that miR-27 is involved in angiogenesis, adipogenesis, oxidative stress, insulin resistance, which are the known processes associated with atherosclerosis. Recent evidence indicates that miR-27b is a strong regulator of lipid metabolism in the human liver via regulating the expression of several of key lipid metabolism-related genes, including ABCA1, LDLR, Angptl3 and Gpam[27, 41]. In addition, the expression levels of miR-27 are closely associated with clinical factors and the prognosis of patients with atherosclerosis obliterans, suggesting that it might serve as a potential biomarker for atherosclerosis[20, 42]. Furthermore, it has been reported that miR-27 has provided a potent atheroprotective function in endothelial cells activated by laminar shear stress[43]. At the same time, our results have shown a positive effect of miR-27 on the pathological process of atherosclerosis through reducing accumulation of intracellular lipids and secretion of pro-inflammatory cytokines in apoE KO mice.

The biological functions of LPL that can be either antiatherogenic or proatherogenic are relevant to its specificity of tissue distribution. In vascular subendothelium, macrophage-derived LPL may contribute to foam cell formation and the progression of atherosclerosis via a nonenzymatic bridging role that modulates the cell surface accumulation and subsequent cellular uptake of modified lipoproteins especially ox-LDL[44, 45]. In addition, suppression of LPL results in the reduced expression of proinflammatory cytokines, such as IL-1β, IL-6, MCP-1 and TNF-α[16]. Our findings were consistent with previously reported effects of macrophage-derived LPL on secretion of proinflammatory cytokines and intracellular lipid levels.

MiRNAs exert their biological effects by targeting the mRNA of multiple target genes. Our group has previously demonstrated that miR-27a/b repressed the expression of endogenous LPL through binding directly to the LPL 3’UTR, and then affected the metabolism of cellular cholesterol in THP-1 macrophages[28]. To fully understand the connection between the miR-27 and atherosclerosis, more studies are required to explore further the potential mechanisms underlying miR-27 effects on lipid metabolism and pro-inflammatory pathways through targeting the LPL gene *in vivo*. ApoE KO mice fed high-fat diet were given a tail vein injection with miR-27 agomir/antagomir or their respective scrambled controls to explore the potential effects of miR-27. Our present study has also revealed miR-27a/b negatively regulates the LPL mRNA and protein expression in aortic lesion areas and peritoneal macrophages of apoE KO mice. Furthermore, miR-27a/b decreased the levels of plasma inflammatory cytokines, consistent with the results in cell culture experiments. Meanwhile, we found miR-27a/b agomir decreased plasma TC and LDL-C levels as well as peritoneal macrophages cholesterol contents compared with control mice. Specifically, systemic treatment with miR-27a/b agomir significantly decreased lesion size and lipid contents in the aorta of apoE KO mice. It is possible that miR-27a/b reduces LPL expression, then inhibits lipid accumulation and secretion of proinflammatory cytokines, and subsequently regulates the development of atherosclerotic lesions in apoE KO mice.

Our previous study has revealed that miR-27a/b attenuated ox-LDL uptake and affected cholesterol constitutes in THP-1 macrophages. Furthermore, we further explored the effect of miR-27a/b on the lipid composition in apoE KO mice, and found that enhancing miR-27a/b function with agomir reduced intracellular TC, CE and FC, but inhibiting miR-27a/b increased those in peritoneal macrophages. The uptake of cholesterol-rich lipoproteins including ox-LDL into the THP-1 macrophages is mostly accomplished through the scavenger receptors on the cell surface, such as SR-A1, LOX-1, CD36 and CXCL16, and plays a central role in foam cell formation[46–48]. Therefore, we measured the scavenger receptor expression to reveal the effect of miR-27a/b on lipid uptake in ox-LDL-treated THP-1 macrophages. Our findings indicated that scavenger receptor expression was suppressed by transfection of cells with miR-27a/b mimic, but increased by transfection with miR-27a/b inhibitor. Nevertheless, miR-27a/b inhibitor-induced effects on mRNA expression of scavenger receptors were dramatically reversed when THP-1 macrophages were transfected with LPL siRNA. These anti-atherosclerotic effects of miR-27a/b may result from miR-27-mediated regulation of the expression of LPL, suggesting a role for scavenger receptors in lipid uptake. However, we measured the effects of miR-27a/b on the expression of only a few scavenger receptors. More studies will be required to elucidate the complete target list of miR-27a/b and fully understand how miR-27a/b affects lipid composition of THP-1 macrophages.

The current study has also revealed that the expression of NF-κB, a key nuclear factor related to inflammatory response, was significantly altered, and secretion of the proinflammatory cytokines was observably suppressed in THP-1 macrophages in response to treatment with miR-27a/b mimic. It is well known that the activity of NF-κB is normally determined by its phosphorylation status and nuclear translocation. We detected the NF-κB protein expression in phosphorylation status by western blot analysis. The results revealed that phosphorylation of NF-κB was reduced in THP-1 macrophages treated with miR-27a/b mimic, suggesting that NF-κB might be involved in the effects of miR-27a/b on atherosclerosis.

**Fig 8:**
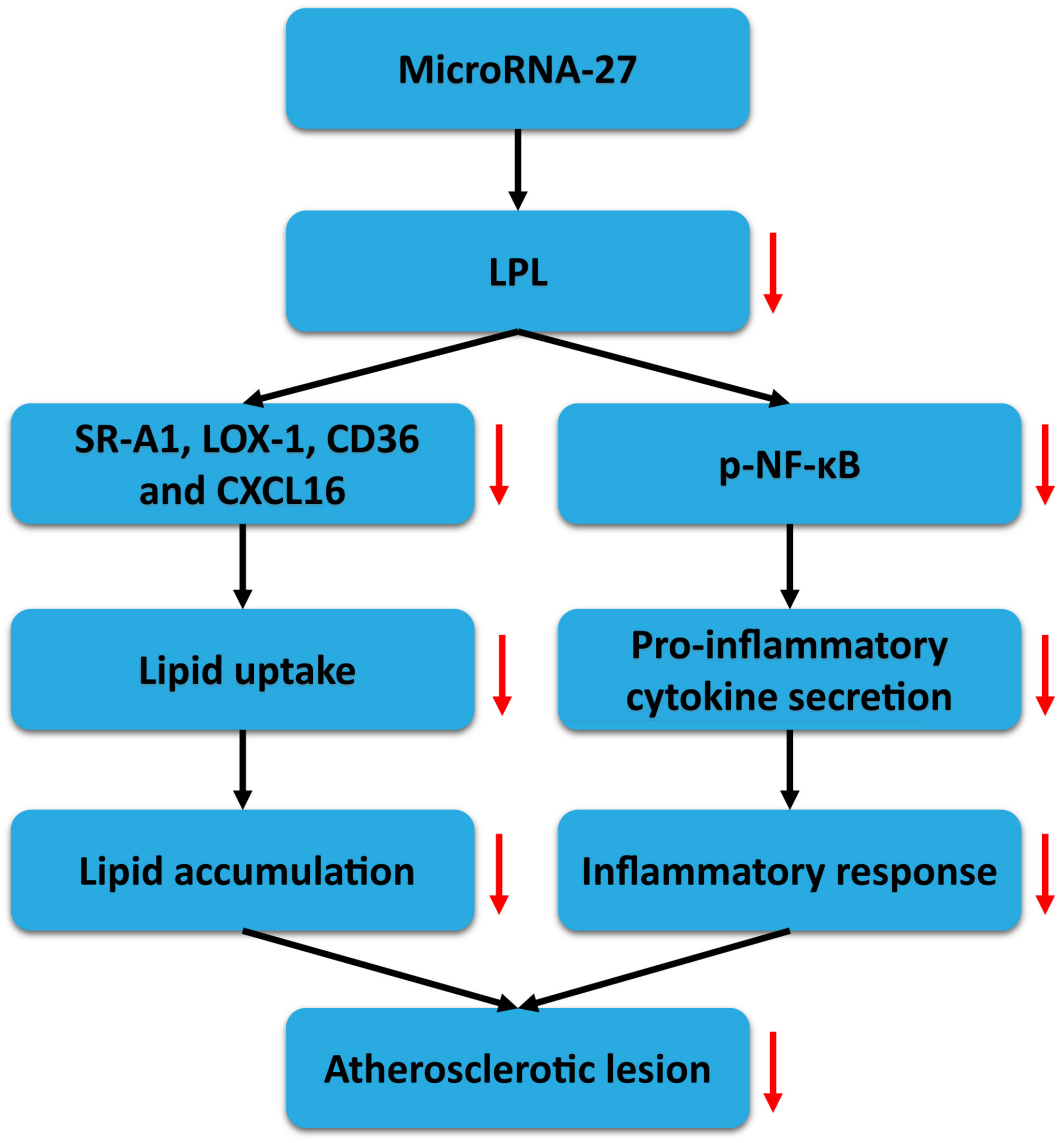
A role for miR-27 in negative regulation of lipid accumulation and proinflammatory cytokine secretion through targeting LPL gene. MiR-27, binding to the LPL 3’UTR to accelerate degradation or repress post-transcriptional expression of mRNA, can inhibit the expression of LPL, and then attenuate lipid accumulation and secretion of proinflammatory cytokines.

In our study, as shown in Fig 8, miR-27 is negatively associated with the expression and activity of LPL through directly targeting the 3’UTR of macrophage LPL, and serves as an important modulator contributing to the macrophage cellular lipid homeostasis and inflammatory response related to atherogenesis. It may be speculated that a novel specific method that effectively regulates miR-27 expression within macrophages might be used to control the development of atherosclerosis. Taken together, our findings may shed new light on the diagnosis and treatment of atherosclerosis.
